# Diagnostic Concordance Using Japan Narrow‐band Imaging Expert Team Classification for Diagnosing Colorectal Neoplasms: A Web‐based Diagnostic Concordance Study

**DOI:** 10.1002/deo2.70232

**Published:** 2025-11-14

**Authors:** Taku Sakamoto, Yasuhiko Mizuguchi, Hideki Ishikawa, Yoshitaka Murakami, Yutaka Saito, The Japan NBI Expert Team (JNET)

**Affiliations:** ^1^ Department of Gastroenterology Institute of Medicine University of Tsukuba Ibaraki Japan; ^2^ Endoscopy Division National Cancer Center Hospital Tokyo Japan; ^3^ Department of Molecular‐Targeting Prevention Kyoto Prefectural University of Medicine Kyoto Japan; ^4^ Department of Medical Statistics Toho University Tokyo Japan

**Keywords:** colorectal neoplasms, endoscopy, image interpretation, narrow band imaging, observer variation

## Abstract

**Objectives:**

The Japan Narrow‐band Imaging Expert Team (JNET) classification is widely used for magnified endoscopic diagnosis of colorectal neoplasms. However, its diagnostic concordance, particularly among the core members who contributed to its development, has not been sufficiently evaluated. Therefore, this study aimed to assess the diagnostic concordance of the JNET classification among JNET core members using a web‐based image interpretation test.

**Methods:**

A total of 27 JNET core members performed a web‐based static image reading test in two separate sessions. Each image was classified according to the JNET criteria, and the diagnostic concordance rate (DCR) was analyzed. Cases were categorized as having high (≥80% consensus), moderate (70%–79% consensus), or low (<70% consensus) agreement. The impact of secondary findings on diagnostic classification was explored for the secondary analysis.

**Results:**

Agreement rates were significantly higher for sessile serrated lesions/hyperplastic polyps (SSL/HP) (>85%) than for neoplastic lesions. In the first session, the DCR for neoplastic lesions was substantially lower, with 54% for low‐grade intramucosal neoplasia, 63% for high‐grade intramucosal neoplasia/T1a, and 52% for T1b. The classification of T1b lesions showed notable variability. Further, while secondary findings influenced classification, this remained an exploratory analysis rather than a primary outcome.

**Conclusions:**

While the JNET classification demonstrated high diagnostic concordance for SSL/HP, variability remained in neoplastic lesions, particularly in T1b cancer. These findings highlight the need for further refinement of the classification system to improve its diagnostic concordance in clinical practice.

## 1 Introduction

Magnified narrow‐band imaging (NBI) is widely used for qualitative diagnosis of colorectal polyps. Based on endoscopic findings, lesions are classified as hyperplastic polyps (HP), sessile serrated lesions (SSL), low‐grade intramucosal neoplasia (LGIN), high‐grade intramucosal neoplasia (HGIN)/shallow submucosal invasive cancer (T1a), or deep submucosal invasive cancer (T1b). The Japan NBI Expert Team (JNET) classification categorizes NBI findings into four types: Type 1, Type 2A, Type 2B, and Type 3, based on presumed histology [1]. The NBI expansion classification, reported by Iwatate et al. [2], serves as the diagnostic foundation, incorporating vessel and surface patterns with high histopathological accuracy.

The JNET classification is based on vessel and surface patterns observed under magnification. However, evaluating the degree of irregularity is challenging due to the minute nature of these findings. Borderline cases often overlap in daily clinical practice, making classification difficult [3]. Thus, diagnostic accuracy alone may not be sufficiently robust. For reliable application in clinical practice, the classification should ensure both high diagnostic accuracy (objectivity) and strong diagnostic concordance.

Nine years have passed since the JNET classification was introduced, with ongoing efforts to align findings from academic conferences and study groups. Therefore, this study aimed to evaluate diagnostic concordance among endoscopists in assessing these findings.

**FIGURE 1:**
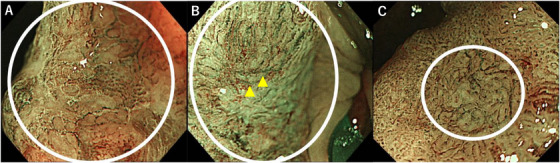
Figure 1 shows representative examples of secondary findings. The circular structures indicate the region of interest (ROI) used to highlight the area where the secondary finding is located. These same images were used during the reading sessions to explain the location of the finding. (A): Scattered vessels, (B): Thick, linearized/ meandering atypical vessels in the tumor (yellow arrow), (C): Demarcated area is defined as a well‐demarcated area with irregular and/or surface pattern wider than approximately 5 mm.

Additionally, Type 3 JNET plays a crucial role in managing neoplastic lesions, and the use of endoscopic treatment is expanding with recent advancements. Ideally, lesions suitable for minimally invasive endoscopic therapy should be identified through magnified observation. As an exploratory analysis, we examined the impact of secondary findings (Figure 1), which are evaluated in addition to the vessel and surface patterns defined in the JNET classification and include features suggestive of deep submucosal invasion, such as scattered vessels, thick linear or meandering atypical vessels, and demarcated areas, from the current classification system on the accuracy of diagnosing T1b.

## 2 Methods

### 2.1 Study Design

This was a web‐based image interpretation study using static magnified endoscopic images of colorectal lesions, conducted among expert endoscopists (JNET core members) in a multi‐institutional setting. Two reading sessions were held (January–March and May–July 2020). The first session used magnified NBI images alone to assess interobserver concordance based solely on the JNET criteria, whereas the second session included sequential white‐light imaging (WLI) and non‐magnified NBI to simulate routine clinical observation. The two sessions were separated to avoid carry‐over effects. Intraobserver concordance was not assessed because the primary aim was to evaluate interobserver concordance and the influence of additional contextual information from WLI.

### 2.2 Participants

A total of 27 JNET core members who were involved in the development of the JNET classification participated in this study. No specific criteria were set for clinical experience, colonoscopy volume, or board certification, as the focus was on agreement among experts familiar with the JNET classification system.

### 2.3 Study Protocol

Each participant evaluated 150 colorectal lesions using magnifying endoscopy. The two reading sessions were conducted at least one month apart to minimize recall bias. To prevent carryover effects, the order of case presentations was randomized between sessions. In the second test, white‐light images, non‐magnified NBI images, and region of interest (ROI) images from the first test were presented sequentially, with the case order reshuffled (Figure S1).

### 2.4 Definition of Diagnostic Concordance Rate

For each case, the diagnostic concordance rate (DCR) was calculated as the proportion of the 27 participating physicians who selected the most frequently chosen JNET classification.

### 2.5 Study Size

This study was exploratory in nature; therefore, no formal statistical sample size calculation was performed. The target of 150 lesions was set a priori on feasibility grounds (reader time burden, anticipated completion rates, and image availability) to ensure that all 27 expert readers could complete both sessions within the scheduled window.

### 2.6 Lesion and Image Selection

Images were captured using a magnifying colonoscope (CF‐H260AZI or PCF‐Q260ZI; Olympus Optical, Japan) and a standard videoendoscopic system (EVIS LUCERA; Olympus, Japan) during colonoscopy examinations at the National Cancer Center Hospital, Tokyo, from January 2015 to March 2017. Magnified images were captured using a moderate‐to‐high‐level power zoom. Endoscopic or surgical resection was performed for all lesions, and histopathological data were available for all observed lesions. Five experienced endoscopists affiliated with the National Cancer Center Hospital, Tokyo, rated 246 lesions that met the above criteria on a 5‐point scale for image quality [4], which also includes the histopathological distribution of these lesions. From these, 150 cases with a mean target of ≥4.5 were selected for the reading study. The clinicopathological information of the selected lesions is shown in Table 1.

**TABLE 1:** Clinicopathological features of lesions.

	Morphology^*^				
	LST‐G	LST‐NG	Sessile	Flat or depressed	Total
HP/SSL, % (*n*)	N/A	N/A	0 (0)	8.7 (13)	13
LGIN % (*n*)	4.7 (7)	7.3 (11)	4.0 (6)	12,7 (19)	43
HGIN or T1a, % (*n*)	10.7 (16)	22.0 (33)	10.0 (15)	4.7 (7)	71
T1b, % (*n*)	3.3 (5)	2.0 (3)	5.3 (8)	4.7 (7)	23
Total	28	47	29	46	150

* Morphology is based on the Paris Classification. Laterally spreading tumors (LSTs) are subclassified into granular type (LST‐G) and non‐granular type (LST‐NG) according to the Japanese classification. LST‐G: laterally spreading tumor, granular type; LST‐NG: laterally spreading tumor, non‐granular type; N/A: not applicable; HP: hyperplastic polyp; SSL: sessile serrated lesion; LGIN: adenoma with low‐grade dysplasia confined to the mucosa; HGIN: adenoma with high‐grade dysplasia or carcinoma in situ (Tis); T1a: superficial submucosal invasive carcinoma; T1b: deep submucosal invasive carcinoma.

### 2.7 Response Items for Reading Test

A confidence level (low or high) was assigned and selected for each of the four JNET classifications. High confidence was defined as >90% certainty of the endoscopist's judgment; low confidence was defined as <90% certainty. The expected histopathological diagnosis was selected from HP or SSL, LGIN (adenoma with low‐grade dysplasia confined to the mucosa), HGIN (adenoma with high‐grade dysplasia or carcinoma in situ [Tis]), or T1a (superficial submucosal invasive carcinoma), and T1b (deep submucosal invasive carcinoma) [1, 2, 5, 6, 7]. Additionally, the presence or absence of the findings listed in the JNET classification was selected as the basis for determining the JNET classification. When Type 2B or Type 3 cancer was selected, the presence or absence of secondary findings and regionality—potential indicators of T1b—were also evaluated (Figure S2).

### 2.8 Assessment

To examine the degree of concordance between examiners and the JNET classification of cases, we focused on the number of examiners who selected the same category as the JNET criteria. Cases were classified as follows:

- High agreement: ≥22 of 27 examiners (agreement rate >80%)
- Moderate agreement: 19–21 of 27 examiners (agreement rate >70%)
- Low agreement: ≤18 of 27 examiners (agreement rate <70%)

We examined the distribution of high, moderate, and low agreement across different lesion types (SSL/HP, LGIN, HGIN, T1a, and T1b)

### 2.9 Significance of Secondary Findings Associated With Deep Submucosal Invasion (Exploratory Analysis)

As an exploratory analysis, we evaluated the potential impact of secondary findings on diagnostic accuracy by analyzing changes in classification for JNET 2B and 3 cases with low confidence. Accuracy was defined as follows:

- JNET 2B‐low with secondary findings → classified as Type 3
- JNET 3‐low without secondary findings → classified as Type 2B

**FIGURE 2:**
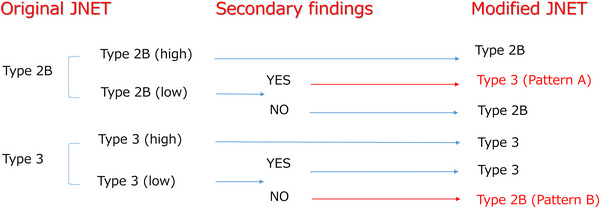
Modification patterns of the original JNET classification according to the presence or absence of secondary findings. In this exploratory analysis, two reclassification patterns were defined: Pattern A, where low‐confidence Type 2B lesions with secondary findings (scattered vessels, thick linear or meandering atypical vessels, or a demarcated area) were reclassified as Type 3; and Pattern B, where low‐confidence Type 3 lesions without secondary findings were reclassified as Type 2B. These two patterns correspond directly to the ROC analysis conditions labeled “A” and “B” in Figure 5. JNET, Japan NBI Expert Team.

Diagnostic accuracy was calculated based on the sensitivity and specificity of pTis‐T1a for JNET 2B and pT1b for JNET 3 (Figure 2).

### 2.10 Ethic Statement

This study was approved by the Institutional Review Board (IRB) of the National Cancer Center Hospital (Approval No: 2018–082) and was registered in the University Hospital Medical Information Network (UMIN) Clinical Trials Registry (UMIN ID: 000039307). The IRB waived the requirement for informed consent for participation in the study. All patients provided written informed consent for the colonoscopy and endoscopic treatment.

## 3 Results

### 3.1 Diagnostic Concordance Rate

**FIGURE 3:**
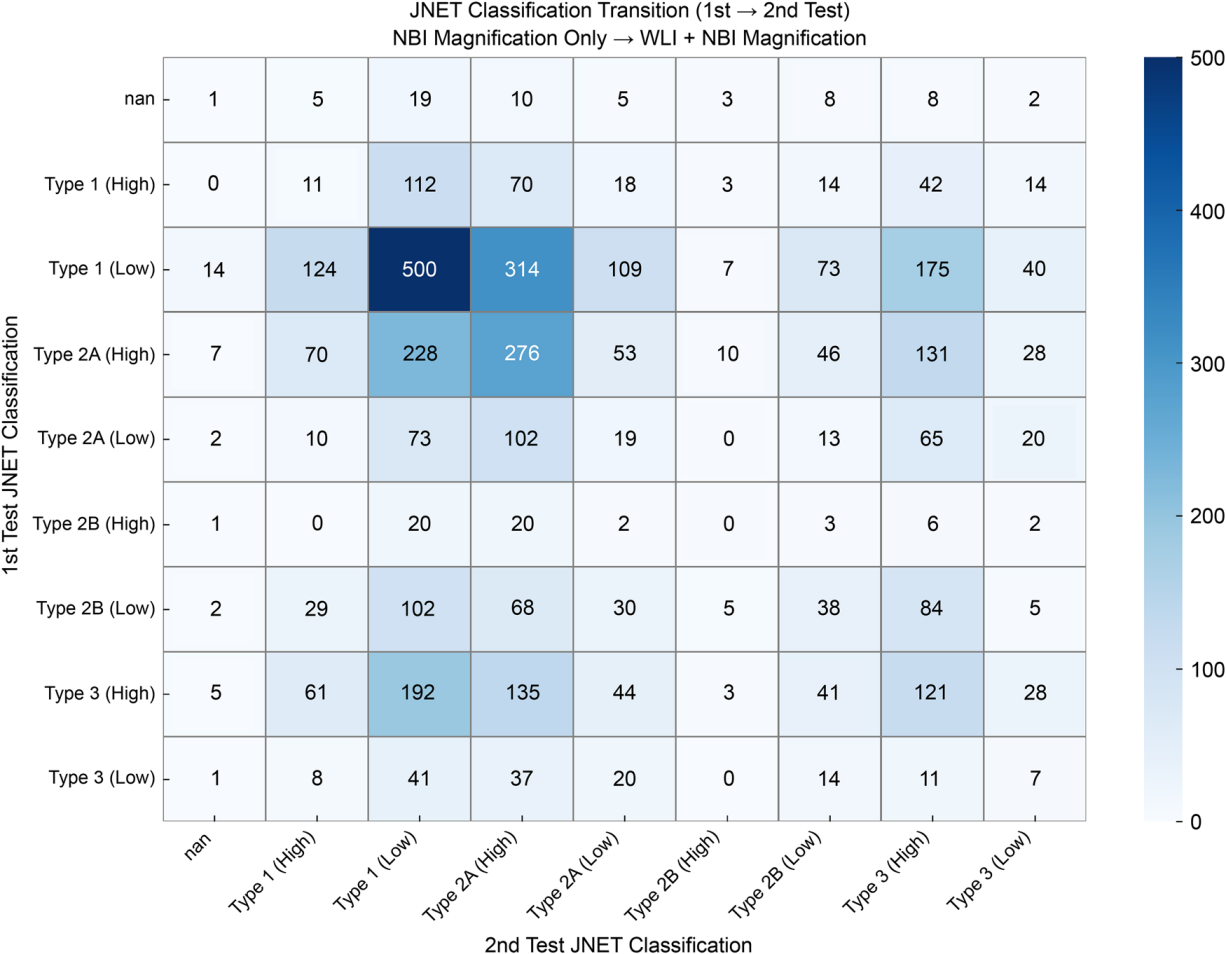
JNET classification transition between the first test (NBI magnification only) and the second test (WLI overview followed by NBI magnification). Each cell shows the number of case–reader combinations assigned to the corresponding JNET category in the first and second sessions. Color intensity reflects the relative frequency, with darker colors indicating higher counts. Notable changes included transitions between Type 2A and Type 2B, which were further quantified in the Results section. The transition from Type 2B to Type 3 suggests that WLI may influence the evaluation of invasion depth. JNET, Japan NBI Expert Team; NBI, narrow‐band imaging; WLI, white‐light imaging.

Table 2 summarizes the DCR among JNET core members. The majority of JNET classifications remained stable between the first and second reading sessions (Table 2 and Figure 3). Type 1 and Type 3 classifications showed high consistency between sessions, while notable reclassifications occurred between Types 2A and 2B, accounting for 7.3% of all case–reader combinations. When viewed within each category, 23.2% of Type 2A cases were reclassified as Type 2B, and 26.9% of Type 2B cases were reclassified as Type 2A. Some low‐confidence Type 2B cases were upgraded to high‐confidence cases. The DCR was high for SSL/HP lesions (>85%) but lower for neoplastic lesions such as low‐grade and high‐grade tumors. The DCR improved from 54% to 70% for LGIN and from 52% to 70% for T1b, but decreased slightly from 63% to 58% for HGIN/T1a. This improvement likely reflects the addition of WLI and non‐magnified NBI in the second session, providing a more comprehensive lesion assessment. Agreement was lower for ESD‐eligible lesions due to difficulty in distinguishing Type 2A and 2B, making this population distinct from standard cases.

**TABLE 2:** Degree of agreement with diagnosis.

	HP/ SSL		LGIN		HGIN/ T1a		T1b		Overall	
	1^st^	2^nd^	1^st^	2^nd^	1^st^	2^nd^	1^st^	2^nd^	1^st^	2^nd^
High, %	85	85	37	47	41	31	35	44	42	42
Moderate, %	92	85	54	70	63	58	52	70	61	65
Low, %	92	92	79	79	80	78	65	91	79	81

High, Moderate, and Low agreement are defined as ≥80%, ≥70%, and <70% concordance, respectively. Each value represents the proportion of cases meeting the corresponding threshold, which were assessed independently rather than as mutually exclusive categories; therefore, the percentages do not necessarily sum to 100%. When confined to neoplastic lesions excluding SSL and HP, the agreement rates were 33%, 58%, and 77% in the first survey and 38%, 64%, and 80% in the second survey, respectively. HP: hyperplastic polyp; SSL: sessile serrated lesion; LGIN: adenoma with low‐grade dysplasia confined to the mucosa; HGIN: adenoma with high‐grade dysplasia or carcinoma in situ (Tis); T1a: superficial submucosal invasive carcinoma; T1b: deep submucosal invasive carcinoma.

### 3.2 JNET Diagnosis by Pathology

**FIGURE 4:**
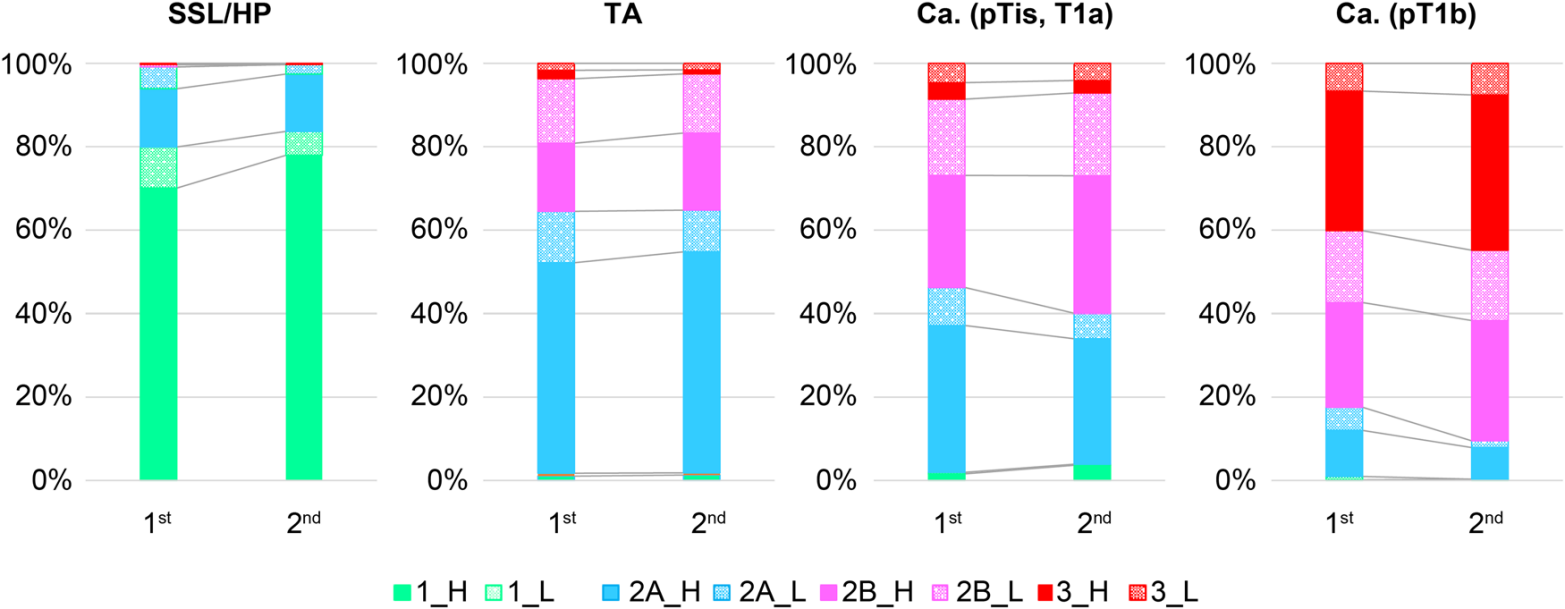
Distribution of JNET classification diagnoses according to histopathological diagnosis in the first and second reading sessions. For each pathological category—HP/SSL, LGIN, HGIN/T1a, and T1b—stacked bars indicate the proportion of lesions classified as JNET Type 1, 2A, 2B, or 3. Data are based on 150 lesions evaluated by 27 expert endoscopists. HGIN, adenoma with high‐grade dysplasia or carcinoma in situ (Tis); HP, hyperplastic polyp; JNET, Japan NBI Expert Team; LGIN, adenoma with low‐grade dysplasia confined to the mucosa; SSL, sessile serrated lesion; T1a, superficial submucosal invasive carcinoma; T1b, deep submucosal invasive carcinoma.

Over 80% of SSL/HP cases were classified as Type 1. Approximately 65% of tubular adenomas were Type 2A, and 30% were Type 2B. For intramucosal or slightly invasive carcinomas, Type 2B diagnoses increased in the second session. Type T1b lesions were mostly classified as Type 2B or Type 3, with the Type 2B‐to‐Type 3 ratio remaining largely unchanged (Figure 4).

### 3.3 Impact of Secondary Findings (Exploratory Analysis)

**FIGURE 5:**
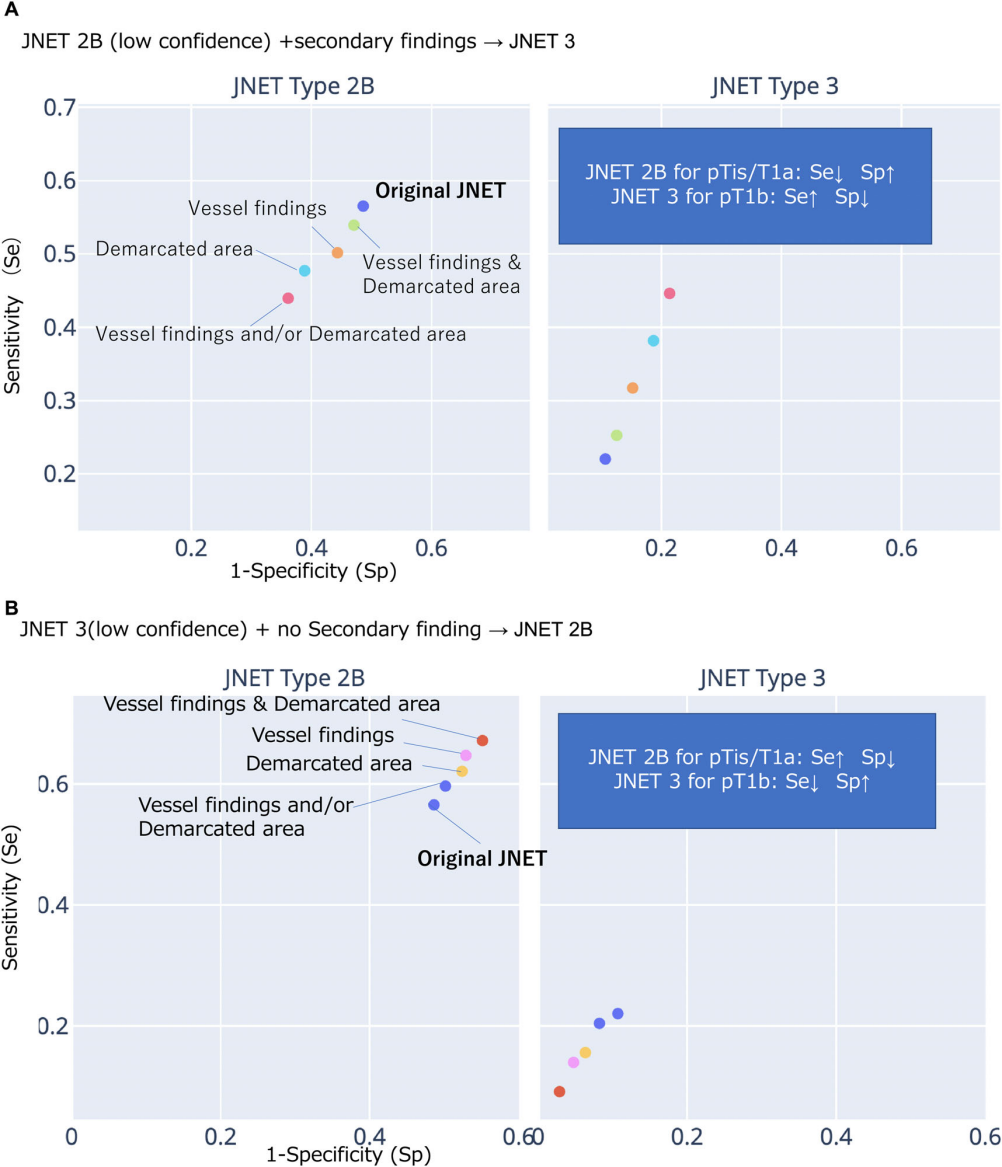
Diagnostic performance for predicting deep submucosal invasion when applying hypothetical reclassification rules to low‐confidence JNET Type 2B or Type 3 lesions in the first reading session using magnifying NBI images only. (A) A condition in which low‐confidence Type 2B lesions with secondary findings suggestive of deep invasion are upgraded to Type 3. The left panel shows lesions originally classified as JNET Type 2B, and the right panel shows lesions originally classified as JNET Type 3, with each panel indicating changes in sensitivity and specificity when secondary findings are incorporated. (B) A condition in which low‐confidence Type 3 lesions without secondary findings are downgraded to Type 2B. The left panel shows lesions originally classified as JNET Type 2B, and the right panel shows lesions originally classified as JNET Type 3, with each panel indicating changes in sensitivity and specificity when secondary findings are incorporated. All classifications represent endoscopic assessments according to the JNET criteria and do not indicate histopathological diagnoses.

In this exploratory analysis, JNET 2B showed 45% sensitivity and 71% specificity, while JNET 3 showed 40% sensitivity and 94% specificity. When 2B‐low‐confidence cases were reclassified as Type 3 cases based on secondary findings, sensitivity dropped slightly to 42%, while specificity increased to 73%. Conversely, reclassifying 3‐low‐confidence cases as 2B cases in the absence of secondary findings improved sensitivity to 46% but reduced specificity to 70%. These findings suggest that secondary findings modestly influence the distinction between Types 2B and 3 cases but do not markedly improve diagnostic accuracy (Figure 5).

## 4 Discussion

This study primarily assessed interobserver agreement in JNET classification rather than its diagnostic accuracy. A significant proportion of cases met ESD selection criteria, making this dataset distinct from routine screening populations. As the study was conducted at a high‐volume center where complex cases are more common, the findings should not be generalized to overall JNET classification accuracy in standard colorectal endoscopy. However, the high agreement in feature selection suggests that JNET classification provides a structured and reliable framework for lesion assessment, even in challenging cases.

Despite high concordance for SSL/HP, variability remained for neoplastic lesions, particularly T1b, highlighting the need for refinement of JNET classification, especially in distinguishing 2B from 3. This may require objective, standardized interpretation, potentially aided by artificial intelligence (AI)‐based systems.

To contextualize the diagnostic agreement observed with JNET classification, it should be compared with previous methods. In Japan, before NBI became widespread, pit pattern diagnosis with magnifying endoscopy was the standard for histopathological estimation [8, 9, 10, 11, 12]. A previous web‐based static image study from our group, which used the same diagnostic framework for pit pattern assessment, demonstrated comparable interobserver concordance. Although direct comparison is limited by differences in datasets and sample size, the diagnostic agreement achieved with JNET appears generally similar to that observed with pit pattern analysis.

In this study, diagnostic concordance improved in the second session, particularly for LGIN and T1b. As the interval between the two sessions was at least one month, and the order of case presentations was randomized, this improvement likely reflects better understanding of classification criteria than short‐term memory effects. In clinical practice, lesions are first detected with WLI and then evaluated in detail with NBI magnification. Thus, assessment relies not only on magnification but also on the overall morphology under WLI. Many lesions, particularly typical LGINs or T1bs, can often be diagnosed without magnification, which may explain the improved consistency in the second session. Although the JNET classification was originally defined based solely on magnified NBI findings, in actual endoscopic practice, endoscopists inevitably interpret the lesion considering its macroscopic morphology observed under white‐light imaging. Particularly for depressed‐type or irregularly shaped tumors, a preconception of higher malignant potential may subconsciously influence the interpretation of microvascular and surface patterns. Therefore, the apparent change in classification between sessions does not imply inconsistency of the JNET system itself but rather reflects how the integration of macroscopic and microstructural information enhances diagnostic consistency under real clinical conditions. Although the integration of macroscopic and microstructural information can enhance diagnostic consistency, subjective variability among individual endoscopists remains an inherent limitation. Therefore, future refinement of the JNET classification should focus on developing objective and standardized interpretation systems. Artificial intelligence–based image analysis has the potential to provide quantitative and reproducible evaluation of microvascular and surface patterns, thereby reducing observer dependency and improving the overall reliability of the JNET classification.

HGIN and T1 lesions often contain mixed components of low‐ and high‐grade atypia, creating a morphological continuum that blurs the distinction between JNET Types 2A and 2B. This overlap likely contributes to the relatively frequent reclassification between these categories. As Type 3 lesions are strongly linked to surgical treatment, diagnostic ambiguity in these borderline categories may lead to overtreatment of lesions amenable to endoscopic resection. Clarifying criteria—such as defining Type 2A by vessels larger in caliber than the surrounding mucosa—may help improve consistency. Furthermore, incorporating secondary findings into the interpretation of low‐confidence Type 3 lesions could modestly increase sensitivity for T1b cancer and prevent unnecessary surgery. However, this remains speculative, and further validation in prospective studies is warranted.

Given these challenges, the diagnostic accuracy for Type 2B remains suboptimal. Even in studies conducted during the establishment of the JNET classification, sensitivity was approximately 45% [2]. Although our study did not primarily evaluate diagnostic accuracy, interpretation based solely on magnified images yielded a 46% accuracy rate, which increased to 55% when overall lesion characteristics were considered. The high transition rates between Types 2A and 2B (23% and 27%) indicate susceptibility to reclassification when additional information, such as WLI or gross morphology, is considered. Thus, judgment depends on both magnified features and macroscopic impressions. Furthermore, because up to 20% of SSLs were classified as Type 2A, clearer differentiation based on vessel caliber relative to surrounding mucosa may improve diagnostic consistency. In addition to the primary analysis of diagnostic concordance, we conducted an exploratory evaluation of secondary findings. Considering the current developments in endoscopic treatment and the oncological characteristics of colorectal cancer, the indications for minimally invasive endoscopic therapy may continue to expand, even in cases with a possibility of T1 cancer [13, 14, 15]. We explored whether incorporating secondary findings into the classification of Types 2B and 3 could enhance accuracy. However, changes in sensitivity and specificity were small, with no clear clinical benefit. These exploratory findings require validation in larger prospective studies.

While the integration of macroscopic and microstructural information can enhance diagnostic consistency, subjective variability among endoscopists remains inevitable. Therefore, future refinement of the JNET classification should focus on establishing objective and standardized interpretation systems. Artificial intelligence–assisted image analysis holds promise for providing quantitative and reproducible assessments of microvascular and surface patterns, thereby reducing interobserver variability. Integrating AI‐based support into the JNET framework may not only improve diagnostic reliability but also facilitate real‐time decision‐making in endoscopic practice.

This study has several limitations. First, the analysis was based on still‐image readings obtained from a single institution. Although moving images would ideally allow for a more dynamic and realistic evaluation, the use of still images with a fixed ROI helped minimize variability in the evaluation site and addressed some of the limitations inherent in video‐based assessments. Second, while the study evaluated interobserver agreement among expert endoscopists, it did not assess intraobserver reproducibility. Moreover, all participating readers were highly experienced in JNET classification, limiting the generalizability of the findings to non‐expert endoscopists. While some studies suggest that structured training can improve diagnostic performance, differences in diagnostic interpretation based on experience remain evident, particularly in challenging or borderline cases [16, 17]. Third, the lesions included in this study were collected from a tertiary referral center specializing in oncology, and many were larger than 10 mm or were borderline cases for which the indication for ESD versus surgical treatment was being considered. Although important for evaluating invasion depth, such lesions often lack clear‐cut‐off features. Thus, assessment of ‘irregularity’ in JNET can be subjective, leading to diagnostic variation. Finally, this study focused on concordance in lesion classification and did not evaluate diagnostic accuracy itself.

Despite these limitations, the study provides important insights into the concordance of the JNET classification among expert endoscopists. High diagnostic concordance for SSL/HP lesions supports the robustness of JNET Type 1, reinforcing its value in screening. However, lower agreement in diagnosing neoplastic lesions, especially T1b cancers, underscores the need for refinements in differentiating Type 2B and Type 3.

Given the observed improvements with sequential evaluation, combining WLI, non‐magnified, and magnified NBI may enhance lesion assessment in practice. Moreover, structured training programs should emphasize the interpretation of secondary findings to improve the detection of deeply invasive cancers. Future prospective studies are required to validate the clinical utility of these findings and further refine the JNET classification for broader use.

In conclusion, the JNET classification demonstrated acceptable interobserver concordance among expert endoscopists, suggesting that it provides a reliable framework for optical diagnosis. However, minor revisions are needed to enhance its robustness and alignment with therapeutic indications. Once a high level of agreement is achieved, the next step is to conduct prospective studies using the Japan Endoscopy Database [18] in daily clinical practice.

## Author Contributions

**Taku Sakamoto**: conceptualization, data curation, formal analysis, and writing—original draft. **Yasuhiko Mizuguchi**: formal analysis and writing—review & editing. **Yoshitaka Murakami**: formal analysis and writing—review & editing. **Hideki Ishikawa**: investigation, formal analysis, and writing—review & editing. **Yutaka Saito**: supervision and writing—review & editing. **The Japan NBI Expert Team (JNET)**: conceptualization, investigation, and writing—review & editing (File S1).

## Conflicts of Interest

The authors declare no conflicts of interest.

## Funding

This work was supported in part by the National Cancer Center Research and Development Funds (grant numbers 29‐A‐13, 2020‐A‐12, and 2023‐A‐15).

## Ethics Statement

**Approval of the research protocol by an Institutional Review Board**: This study was approved by the Institutional Review Board (IRB) of the National Cancer Center Hospital (Approval No: 2018–082) and was conducted in accordance with the ethical standards of the Declaration of Helsinki (as revised in 2013).

## Consent

The IRB waived the requirement for informed consent for participation in this reading study. All patients provided written informed consent for the colonoscopy and endoscopic treatment, including the use of endoscopic images for research purposes.

## Clinical Trial Registration

This study was registered in the University Hospital Medical Information Network (UMIN) Clinical Trials Registry (UMIN ID: 000039307).

## Supporting information




**FIGURE S1**: Reading flow of the examination.
**FIGURE S2** Response items for the reading test.

**FILE S1**: Additional details, including a list of contributing JNET members who participated in the study but are not listed as co‐authors.

## Data Availability

The data supporting the findings of this study are available in the UMIN Clinical Trials Registry under the registration number UMIN000039307. The dataset can be accessed online at https://www.umin.ac.jp/ctr/. Researchers interested in accessing the dataset should refer to the UMIN registry for further details. Additional information regarding data access may be available upon reasonable request to the corresponding author.
