# Supplementary material for: Diagnostic Concordance Using Japan Narrow‐band Imaging Expert Team Classification for Diagnosing Colorectal Neoplasms: A Web‐based Diagnostic Concordance Study
Source: DEN Open. 2025 Nov 14;6(1):e70232. doi: 10.1002/deo2.70232 (PMC12616501; doi:10.1002/deo2.70232)
Supplement: Supplementary file 1 — FIGURE S1: Reading flow of the examination. FIGURE S2 Response items for the reading test. [file DEO2-6-e70232-s002.docx]

**Supplementary Figures**


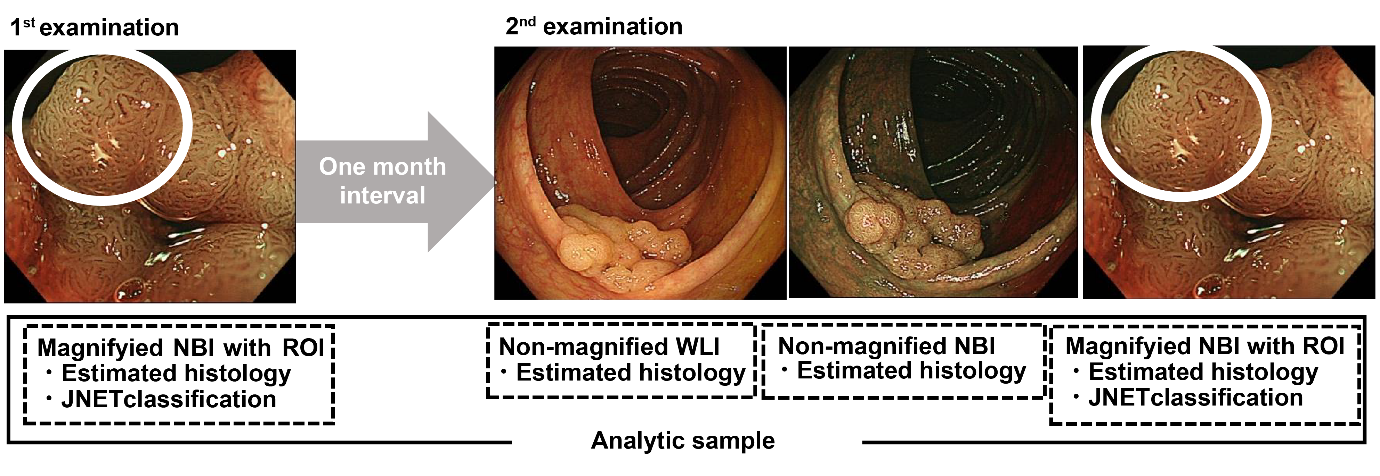
 **Supplementary Figure 1.** Reading flow of the examination


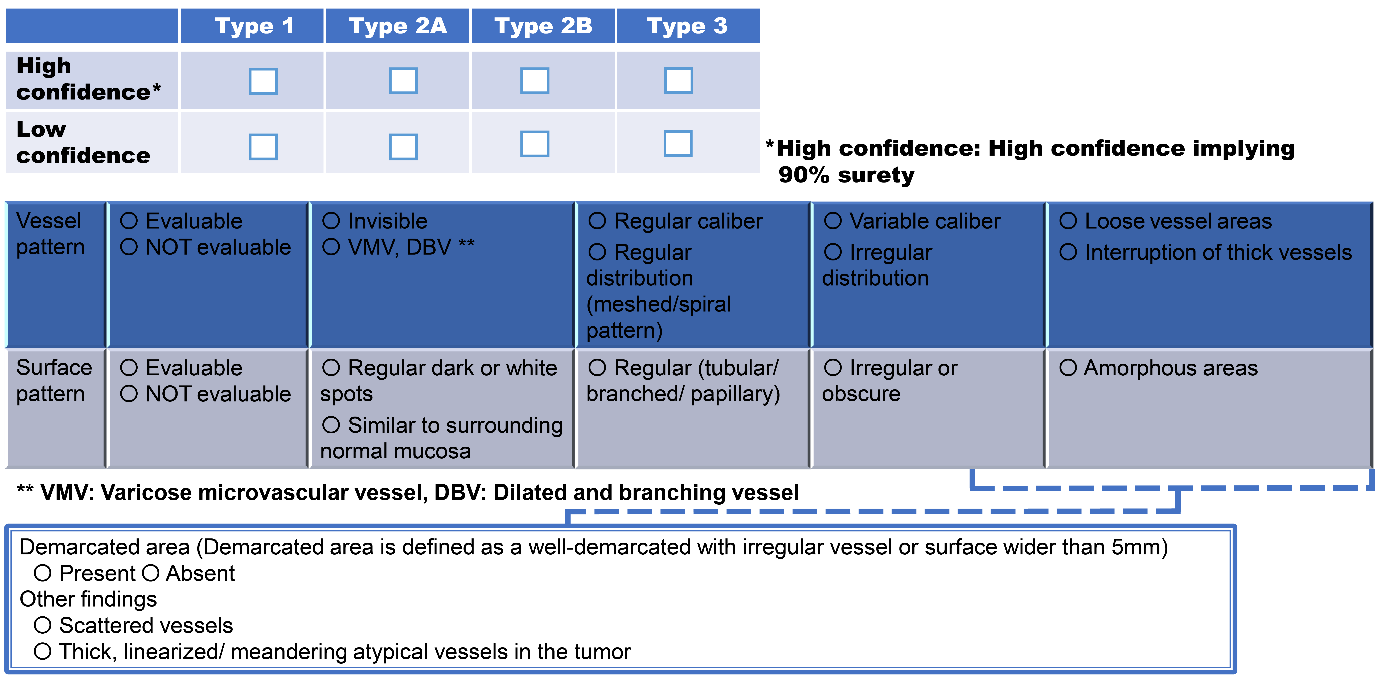


**Supplementary Figure 2.** Response items for the reading test
