# Supplementary material for: Diagnostic Concordance Using Japan Narrow‐band Imaging Expert Team Classification for Diagnosing Colorectal Neoplasms: A Web‐based Diagnostic Concordance Study
Source: DEN Open. 2025 Nov 14;6(1):e70232. doi: 10.1002/deo2.70232 (PMC12616501; doi:10.1002/deo2.70232)
Supplement: Supplementary file 2 — FILE S1: Additional details, including a list of contributing JNET members who participated in the study but are not listed as co‐authors. [file DEO2-6-e70232-s001.docx]

# Supplementary File 1: Contributing Members of the JNET

The following members of the Japan NBI Expert Team (JNET) contributed to this research by proposing the theoretical framework for the study, writing and proofreading the manuscript, and collecting data.

**Hiroaki Ikematsu** (Department of Gastroenterology and Endoscopy, National Cancer Center Hospital East)

**Hirohisa Machida** (Machida Gastrointestinal Hospital)

**Hiroshi Kashida** (Department of Gastroenterology and Hepatology, Kindai University Faculty of Medicine)

**Hiroshi Kawano** (St.Mary's Hospital)

**Hiro-o Yamano** (Department of Gastroenterology and Hepatology, Sapporo Medical University School of Medicine)

**Ichiro Hirata** (Department of Gastroenterology, Osaka Central Hospital)

**Kinichi Hotta** (Shizuoka Cancer Center)

**Kuang-I Fu** (Department of Endoscopy, Kanma Memorial Hospital)

**Masakatsu Fukuzawa** (Tokyo Medical University, Gastroenterology and Hepatology)

**Masashi Misawa** (Digestive Disease Center, Showa University Northern Yokohama Hospital)

**Mineo Iwatate** (Gastrointestinal Center and Institute of Minimally Invasive Endoscopic Care, Sano Hospital)

**Naohisa Yoshida** (Department of Molecular Gastroenterology and Hepatology, Kyoto Prefectural University of Medicine, Graduate School of Medical Science)

**Naoko Nakano** (Department of Gastroenterology, Fujita Health University)

**Naoto Sakamoto** (Sakamoto Endoscopy Clinic)

**Naoto Tamai** (Department of Endoscopy, The Jikei University School of Medicine)

**Nana Hayashi** (Department of Gastroenterology, Hiroshima University Hospital)

**Nozomu Kobayashi** (Endoscopy Division, National Cancer Center Hospital)

**Osamu Turuta** (Department of Gastroenterology, St. Mary's Hospital)

**Shoichi Saito** (Department of Lower GI, Cancer Institute Hospital of Japanese Foundation for Cancer Research)

**Shin-ei Kudo** (Digestive Disease Center, Showa University Northern Yokohama Hospital)

**Shinji Tanaka** (JA Onomichi General Hospital)

**Shigeaki Yoshida** (CEO, Aomori Prefectural Hospital Administration)

**Shiro Oka** (Department of Gastroenterology, Hiroshima University Hospital)

**Takeshi Nakajima** (Department of Genetic Oncology, Osaka International Cancer Institute)

**Takeshi Terai** (Terai Clinic)

**Takahisa Matsuda** (Division of Gastroenterology and Hepatology, Toho University Omori Medical Center)

**Takahiro Fujii** (Takahiro Fujii Clinic)

**Takahiro Horimatsu** (Institute for Advancement of Clinical and Translational Science, Kyoto University Hospital)

**Toshio Uraoka** (Department of Gastroenterology and Hepatology, Gunma University Graduate School of Medicine)

**Toshihiro Kusaka** (Gastroenterology, Senshunkai Hospital)

**Yasushi Sano** (Gastrointestinal Center, Sano Hospital)

**Yoshiki Wada** (Wada Clinic)

**Yoji Takeuchi** (Department of Gastroenterology and Hepatology, Gunma University Graduate School of Medicine)
